# HPV prevalence and distribution characteristics in postmenopausal women from Nanjing, China

**DOI:** 10.1186/s12905-024-02904-8

**Published:** 2024-01-24

**Authors:** Xiaorui Yin, Chunxue Zhang, Xiaoqing Wu, Jing Feng, Jingyan Xie, Yujuan Li

**Affiliations:** Department of Gynecology, Nanjing First Hospital, Nanjing Medical University, No.68, ChangLe Road, Nanjing, 210006 China

**Keywords:** HPV infection, Cervical cancer, Postmenopausal women, Genotypes, Chinese women

## Abstract

**Background:**

Cervical cancer is strongly associated with human papillomavirus (HPV) infection. In this retrospective study, we analyzed the data of postmenopausal women who were tested for HPV in Nanjing First Hospital from 2019 to 2021.

**Methods:**

We retrospectively analyzed the data of 14,608 postmenopausal women aged 45–90 years, who underwent HPV examination in Nanjing First Hospital between January 2019 and December 2021. All participants were tested for 23 HPV genotypes. We subsequently analyzed the infection rate and evaluated the distribution of HPV using the chi-square test.

**Results:**

Our results showed that the HPV infection rate in postmenopausal women in Nanjing, China was 22.36%. In terms of age group, the infection rate was 19.54%, 24.30%, 26.58%, and 14.99% in those aged ≤ 50, 51–60, 61–70, and ≥ 71 years, respectively. The most common HPV subtypes were HPV52 (22.1 3%), HPV58 (15.86%), HPV53 (14.17%), HPV16 (12.61%), and HPV81 (11.66%), in that order. The single-HPV infection rate was 14.23%, and the multiple-genotype infection rate was 8.14% (1189/14,608).

**Conclusions:**

This study showed that in Nanjing, China, the different age groups of post-menopausal women could have different rates of HPV infection, and the most common types were HPV52, HPV58, HPV53, HPV16 and HPV81. These findings highlighted the importance of understanding the epidemiology of HPV infection in specific populations, such as postmenopausal women in Nanjing, China. The results could provide valuable information for healthcare professionals and policymakers to develop targeted prevention and screening strategies for reducing the burden of HPV-related diseases in this population.

## Background

Human papillomavirus (HPV) is a spherical DNA virus, which can cause sexually transmitted diseases. Infection with HPV may occur through many methods, including sexual transmission, close contact, indirect contact, iatrogenic infection, and mother-to-child transmission. Studies have identified more than 200 types of HPV, which can be classified as either high- or low-risk, according to their carcinogenic ability [1]. High-risk factors for HPV infection include a large number of sexual partners; early cohabitation age; young primiparity age; suppressed and altered immune status; and hormone influence [2]. In the high-risk HPV group, HPV 16 and HPV 18 are the most common and have the highest cancer-causing ability. Studies have shown that 50–70% and 7–20% of cases were caused by HPV16 and HPV 18, respectively [3, 4].

HPV infection is very common worldwide, and most women will be infected with HPV in their lifetime [5]. Although most infections in female patients are asymptomatic and transient, persistent infection occurs in some patients, which may cause low-grade or high-grade cervical intraepithelial neoplasia and cervical cancer [6].

Cervical cancer is the second most common cancer in women worldwide, and almost 100% of women are infected with HPV at some point in their lifetime [7]. The risk factors of cervical cancer include multiple births, smoking, immunosuppression, malnutrition, and HPV infection. Among them, the most critical risk factors for cervical cancer are persistent HPV infection and lack of effective screening [8]. Furthermore, HPV is not only a carcinogen of cervical cancer, but also increases the risk of some vulva, vagina, penis, head and neck cancers [9].

The distribution of HPV genotypes shows regional differences [10, 11]. In the Asian population, HPV 18, 52, and 58 are more common. A study in Hunan, China showed that the HPV infection rate was approximately 10.16%, among which HPV16 was the most common sub-type, with an infection rate of 2.19% [12].

Another study on Uyghur women showed that the overall prevalence rate of HPV was 8.83%, for which the prevalence rate of high-risk HPV was 7.25%, and that of low-risk HPV was 1.58%. In order of infection rate, the five most common HPV subtypes are HPV16, HPV 51, HPV 31, HPV39, HPV 58 [13]. According to one survey, the estimated prevalence of HPV infection in women worldwide is 10.5% but varies widely across populations and geographic regions [14].

After menopause, immunity declines and the vaginal microecology changes, which increases the risk of HPV infection. Furthermore, in post-menopausal women, infections are more likely to develop into persistent ones, which could lead to cervical cancer. Simultaneously, as the population continues to age, the number of menopausal women increases and the number of patients with cervical cancer among older women has increased accordingly [15].

In this study, we assessed the distribution characteristics of HPV in postmenopausal women who underwent screening at Nanjing First Hospital from January 2019 to December 2021, to provide a theoretical reference for the cervical cancer screening strategy in postmenopausal women in China.

## Methods

### Target population

In this study, we retrospectively analyzed the data of 14,608 postmenopausal women aged 45–90 years, who underwent HPV examination in Nanjing First Hospital from January 2019 to December 2021. The exclusion criteria were total hysterectomy; systemic infection or autoimmune diseases; surgery for uterine diseases within 3 days; or other cancer types. Ethics approval was obtained from the Ethics Committee of Nanjing First Hospital, Nanjing Medical University (approval number: KY20210604-05). The need for obtaining informed consent was waived by the Institutional Review Board of Nanjing First Hospital, Nanjing Medical University due to the retrospective nature of the study.

### HPV genotyping

HPV deoxyribonucleic acid (DNA) typing was performed using an HPV genotyping kit (Rapid HPV Genotyping MacroArray; Hybribio Ltd., Hong Kong). The assay was based on DNA amplification with HPV L1 consensus primers using polymerase chain reaction and the flow-through hybridization technique. It could recognize 23 HPV genotypes, including 17 high-risk HPV (HR-HPV) sub-types (HPV 16, 18, 31, 33, 35, 39, 45, 51, 52, 53, 56, 58, 59, 66, 68, 73, and 82) and 6 low-risk HPV (LR-HPV) sub-types (HPV 6, 11, 42, 43, 44, and 81). All protocols were performed according to the supplier’s manual, as previously described [16]. The sensitivity and specificity of the Hybribio HPV test kit, high risk-HPV and HPV 16/18 for cervical precancerous lesions screening were 95.1% (95% confidence interval [CI]: 88.1–98.1%) and 87.6% (95%CI: 86.9–88.2%) and 65.9% (95%CI: 55.1–75.2%) and 97.8% (95%CI: 97.5–98.1%), respectively [17].

### Statistical analysis

The Chi-square test was used to compare the difference of HPV infection rate among different groups. A *p*-value of < 0.05 was considered statistically significant. The software SPSS version 25.0 was used for all statistical data analyses (SPSS, IBM, NY, USA).

## Results

### HPV prevalence

Table 1 presents the distribution of HPV infection in all the defined age groups. Of the 14,608 women enrolled, 3,267 tested positive for HPV. In terms of age group, the infection rate was 19.54% (1118), 24.30% (1467), 26.58% (585), and 14.99% (97) in those aged ≤ 50, 51–60, 61–70 and ≥ 71 years, respectively. These results revealed that the HPV infection rate in the 61–70-year-old population was higher than that in other populations. Statistical analysis revealed significant differences between the different age groups (*P* = 0.000, Table 1), indicating that people aged 61–70 were more susceptible to HPV infection.

**Table 1:** Distribution of HPV infection in each age group

Age groups (y)	HPV (n)	Total (N)	n/N
≤ 50	1118	5722	0.195386229
51–60	1467	6038	0.242961245
61–70	585	2201	0.265788278
≥ 71	97	647	0.14992272
Total	3267	14,608	

HPV, human papillomavirus

### Genotype distribution in the HPV positive population

As presented in Table 2, the most common sub-types of HPV infection were HPV52 (22.13%), HPV58 (15.86%), HPV53 (14.17%), HPV16 (12.61%), and HPV81 (11.66%). Statistical analysis revealed significant differences between age groups in terms of HPV52 (*P* = 0.013), HPV58 (*P* = 0.004), and HPV53 (*P* = 0.002). The rates of HPV16 (*P* = 0.166) and HPV81 (*P* = 0.805) infection did not show differences among the different age groups.

**Table 2:** Distribution of different types of HPV in each age group

Age groups (y)	≤ 50	51–60	61–70	≥ 71	Total
HPV	1118	1467	585	97	3276
HPV16	139	173	82	18	412
HPV18	49	64	25	4	142
HPV31	45	73	40	5	163
HPV33	66	88	47	5	206
HPV35	12	29	13	1	55
HPV39	92	110	51	7	260
HPV45	34	55	24	3	116
HPV51	61	85	44	5	195
HPV52	221	322	154	26	723
HPV53	122	230	97	14	463
HPV56	39	71	53	10	173
HPV58	156	227	111	24	518
HPV59	38	72	33	6	149
HPV66	36	56	15	6	113
HPV68	65	90	49	6	210
HPV73	8	15	11	1	35
HPV82	29	29	15	2	75
HPV11	25	30	14	4	73
HPV42	96	140	60	9	305
HPV43	72	95	28	3	198
HPV44	116	133	34	14	297
HPV6	27	57	15	3	102
HPV81	128	178	66	9	381

HPV, human papillomavirus

### Prevalence of single- and multiple-HPV infection

Among the 14,608 women, 3267 tested positive for HPV, of whom 2,078 had a single sub-type of HPV, and 1,189 had multiple sub-type infections (Table 3). The single-HPV infection rate was 14.23% (2078/14,608). Furthermore, we found a significant difference between the different age groups (*P* = 0.000, Table 3). The multiple sub-type of infection rate was 8.14% (1189/14,608), with significant differences between different age groups (*P* = 0.000, Table 3).

**Table 3:** Prevalence of single- and multiple-HPV infections

Age groups (y)	≤ 50	51–60	61–70	≥ 71	Total
Total	5722	6038	2201	647	14,608
HPV	1118	1467	585	97	3267
Single HPV	760	929	334	55	2078
Multiple HPV	358	538	251	42	1189

HPV, human papillomavirus

### Prevalence of high- and low-risk HPV infection

Among the 3267 women who tested positive for HPV, 2717 women were infected by at least one sub-type of HR-HPV, and 550 had multiple infections (Table 4). Overall, the HR-HPV infection rate was 18.60% (2717/14,608) and the LR-HPV infection rate was 3.76% (2717/14,608). Statistical analysis showed that the HR-HPV infection rate was higher than the LR-HPV infection rate in all age groups, and individuals aged 61–70 were significantly more likely to be infected with HR-HPV (*P* = 0.000, Table 4).

**Table 4:** The prevalence of high-risk HPV and low-risk HPV infection

Age groups (y)	≤ 50	51–60	61–70	≥ 71	Total
Total	5722	6038	2201	647	14,608
HPV	1118	1467	585	97	3267
High-risk HPV	892	1212	529	84	2717
Low-risk HPV	226	255	56	13	550

HPV, human papillomavirus

### Prevalence of single- and Multiple-HPV infection of different sub-types

As shown in Table 5, among the women infected with HPV16, 51.21% had single-HPV infection and 48.79% had multiple-HPV infection. The single-HPV infection rates of HPV 52 (48.41%), HPV58 (44.98%), HPV53 (42.76%), HPV81 (44.62%), HPV18 (35.21%) were lower than those of multiple-HPV infection. Statistical analysis showed that the single-HPV infection rate of HPV16 was significantly higher than the other HPV genotypes (*P* = 0.009, Table 5).

**Table 5:** Prevalence of Single- and Multiple-HPV infections of different sub-types

	Single-HPV infection	Multiple-HPV infection	Total infection
HPV52	350	373	723
HPV58	233	285	518
HPV53	198	265	463
HPV16	211	201	412
HPV81	170	211	381
HPV18	50	92	142

HPV, human papillomavirus

## Discussion

Cervical cancer is one of the most common gynecological malignancies. It is one of the leading causes of cancer death in women worldwide. The World Health Organization has set a global goal of eliminating cervical cancer, defined as achieving an incidence of less than 4 cases per 100,000 women per year. The ‘90-70-90’ goal has also been set, defined as follows: 90% of girls should be fully vaccinated against human papillomavirus (HPV) vaccine by age 15, 70% of women should undergo screening with high performance tests by the age of 35 years, and again by the age of 45 years, and 90% of women with cervical disease should receive appropriate treatment [18].

Almost all cervical cancers are caused by persistent HPV infections. Numerous studies have shown that the most common HPV sub-types were HPV16, HPV18, HPV52 and HPV58. Older women, particularly pre- and post-menopausal women, exhibit decreases in immune capability, which results in a weakened ability to clear previous and new HPV infections, which is also reflected in the high HPV infection rate.

In a previous study examining women in Wuhan, the total HPV infection rate was found to be 13.10%, with most women being infected with a high-risk sub-type. There was no significant difference in the prevalence of HPV infection between women before and after menopause. However, the prevalence of high-risk sub-types of HPV was higher in those older than 65 years [15]. In this study, among the total 14,608 women evaluated, 3,267 tested positive for HPV: 22.36% of postmenopausal women had HPV infection. The HPV infection rate in this study was higher than that reported in previous studies [12], which may have been related to the postmenopausal population being more susceptible to HPV infection. In addition, since cervical screening is performed more frequently, an increasing number of individuals in the community could undergo screening. When the results of cervical screening are abnormal, patients are transferred to a higher level of care for further treatment. Nanjing First Hospital is a tertiary referral hospital, which may treat more people with abnormal cervical screenings, leading to a higher infection rate in this research.

Our analysis further showed that the most common HPV sub-types in postmenopausal women were HPV52, HPV58, HPV53, and HPV16. Among them, HPV52 accounted for 22.13% of all infections. HPV16, HPV52, and HPV58 were more likely to occur in people ≥ 71 years old, while HPV53 was more likely to occur in those aged 61–70 years. This inconsistency may have been caused by regional differences and different pathogenicities of different HPV types.

Multiple HPV infections had a higher correlation with high-grade cervical squamous intraepithelial lesions, and their duration was longer than that of single HPV infection [19]. This may have been due to the change in immune status and increase in viral load [10]. In our study, the single HPV infection rate was 14.23%, which is greater than the multiple HPV infection rate of 8.14%. Single HPV infections were more common in people aged from 51 to 60, while multiple HPV infections were more common in people aged from 61 to 70. Studies have demonstrated that multiple HPV infections could increase the incidence of cytological abnormality compared with single HPV infection [20]. Multiple HPV infections could lead to prolonged persistent infection. In addition, patients with multiple high-risk viral loads had a 4 to 6-fold higher risk in cervical precancerous cytology than those with a single high-risk viral load [21]. As seen from Table 5, the HR-HPV infection rate (18.60%) was higher than those of only LR-HPV infection rate (3.76%). The HR-HPV infection rate was higher than the LR-HPV infection rate in every age group. Furthermore, people aged from 61 to 70 were more susceptible to HR-HPV. Among the entire cohort, the HPV infection rate (26.58%, 585/2201) and HR-HPV infection rate (24.03%, 529/2201) were the highest in women aged 61–70 years, followed by 51–60 years old people (24.30%, 1467/6038; 20.07%, 1212/6038). The HR-HPV infection rate was higher than the rate in Beijing found in a previous study, which may be because the examined population in Beijing comprised all people who underwent free screening [22].

Additionally, some postmenopausal women may wish to preserve their fertility after being diagnosed with cervical or other gynecologic malignancies because of personal, cultural, religious, or family considerations [23]. Although it is known that postmenopausal people are more likely to be infected with HPV, there have been very few studies evaluating HPV infection specifically in postmenopausal women. Persistent infection with high-risk HPV could increase the risk of cervical cancer, vagina cancer, and vulvar cancer in postmenopausal women. These cancers typically occur after menopause and are difficult to detect due to the atrophy of cervical tissues. Furthermore HPV persistence is one of the most important factors predicting the risk of CIN2 + recurrence. The risk of CIN2 + recurrence increased with the increase of HPV persistence for up to 1 year. The persistence of HPV after the first year does not appear as a risk factor [24]. Therefor further recommendations have to take into account this feature. Attempts are needed to better categorize patients with HPV infection, thus providing useful information for prognostications and tailoring the most appropriate surveillance [25].

The study found variations in HPV infection rates among different age groups of postmenopausal women. This information is valuable as it helps identify specific cohorts that may require more vigilant screening and monitoring for cervical cancer. For example, the higher infection rates observed in the 61–70 age group might suggest the need for increased attention and targeted interventions in this age range.This information can guide developing more effective and targeted screening strategies, such as recommending more frequent screening or incorporating HPV testing into routine screening protocols for this specific population.

Cervical cancer incidence declines with further promotion of cervical screening. Therefore, it is necessary to promote cervical screening among postmenopausal people and provide timely treatment.

## Conclusions

This study sought to provide valuable insights into the epidemiology of HPV infection in this specific population. Our findings showed that the HPV infection rate differed between the different age groups, and the most common types were HPV52, HPV58, HPV53, HPV16 and HPV81. These results indicated that more attention should be paid to cervical cancer screening to achieve the goal of eliminating cervical cancer.

The study was conducted at a single hospital, which may have introduced a potential source of bias due to the specific characteristics of the patient population in that particular healthcare facility. Therefore, the findings may not fully represent the HPV prevalence and distribution characteristics in postmenopausal women in the broader population. However, by providing data on HPV prevalence and distribution in Nanjing, the study can contribute to informing public health authorities and policymakers in the region about the current state of HPV infection. The study contributes to the body of scientific literature on HPV epidemiology, particularly by focusing on postmenopausal women in a specific geographical region. Our findings can provide information for the benefit of healthcare practices, screening guidelines, and interventions for reducing the burden of cervical cancer in this population. This information can help in developing targeted prevention and screening strategies to reduce the incidence of HPV-related diseases among postmenopausal women.

## Data Availability

The datasets used and/or analysed during this study are available from the corresponding author upon reasonable request.

## Abbreviations

HPV: Human papillomavirus
CIN: Cervical intraepithelial neoplasia
HR-HPV: High-risk human papillomavirus
LR-HPV: Low-risk human papillomavirus
